# Axillary metastases after port site recurrences of gallbladder carcinoma: a case report

**DOI:** 10.1186/s12957-020-01822-x

**Published:** 2020-04-07

**Authors:** Jorieke J. H. T. Nijhuis, M. R. Frederiek Bosscher, Mike S. L. Liem

**Affiliations:** Department of Surgical Oncology, Medisch Spectrum Twente, Enschede, the Netherlands

**Keywords:** Cholecystectomy, Port site, Gallbladder carcinoma, Axilla, Metastasis, Lymphatic metastasis

## Abstract

**Background:**

Incidental gallbladder carcinoma is often discovered after elective laparoscopic cholecystectomy for cholecystitis or cholecystolithiasis. Port site recurrences may occur. Patients with port site metastases of gallbladder carcinoma have a poor prognosis.

**Case presentation:**

A 61-year-old man underwent an elective laparoscopic cholecystectomy because of cholecystitis and gallstones. Pathology revealed a gallbladder carcinoma. After referral to a tertiary center, radical re-resection followed.

Three years later, an epigastric port site recurrence emerged, partially fixed to the xiphoid process. A wide abdominal wall resection was performed, including part of the xiphoid process. Follow-up was continued with periodical imaging and standard blood work.

Three years after resection of this port site metastasis, the patient presented with an occasionally painful mass in the left axilla. Pathology revealed the presence of an adenocarcinoma, most likely arising from the prior gallbladder carcinoma.

Given the extensive dissemination and limited symptoms in the axillary node, we decided against a surgical intervention, instead of adopting a wait-and-see policy. Disease progression occurred within 1 year, and the patient was treated with palliative radiotherapy, followed by palliative chemotherapy.

The patient died of metastatic disease approximately 6.5 years after the initial cholecystectomy.

**Conclusions:**

Port site recurrences of (incidental) gallbladder carcinoma occur after laparoscopic cholecystectomy, despite preventive perioperative measures. Patients with port site recurrences can develop axillary lymph node metastases, similar to other truncal malignancies. Surgical interventions should be limited.

## Background

Gallbladder carcinomas are often incidentally discovered after elective laparoscopic cholecystectomy for the treatment of cholecystitis or cholecystolithiasis. The rate of incidental gallbladder carcinomas is 0.3–1.0% [1]. Patients with stage T1b and T2 carcinomas have a fair prognosis when a radical cholecystectomy (resection of the gallbladder and liver segments IVb and V combined with regional lymph node dissection) is performed [2, 3]. However, port site recurrences are relatively frequent following the laparoscopic resection of the gallbladder, independent of tumor stage [1, 4]. Even for patients with an initially favorable low-stage tumor, port site recurrences are generally regarded as a stage IV disease with a short life expectancy [5].

In this report, we describe the case of a patient who was treated for a gallbladder carcinoma and a subsequent port site recurrence, and who developed upper truncal lymph node metastases years after the initial treatment for the gallbladder carcinoma.

### Case presentation

A 61-year-old white male with no past medical history underwent a laparoscopic cholecystectomy after an episode of cholecystitis and radiologically proven gallstones. The gallbladder was removed through the epigastric port using an endobag and there were no complications. However, the pathology report revealed a moderately differentiated adenocarcinoma (T2).

Subsequently, the patient was referred to a hepatobiliary center and, 3 months after the initial cholecystectomy, a radical cholecystectomy was performed. The initial port sites of the previous cholecystectomy were not resected because an endobag was used during the initial procedure. The pathology report showed no tumor in the secondary resection, and the lymph nodes tested negative for metastases. Follow-up was continued with periodical checkups and standard blood work (with carbohydrate antigen (CA) 19.9).

Three years later, the patient reported symptoms of discomfort and pain around the epigastric port site scar. CA 19.9 level was normal (reference value < 37U/ml). An epigastric port site recurrence was subsequently discovered, partially fixed to the xiphoid process. A wide abdominal wall resection was performed, including part of the xiphoid process, with the reconstruction completed using a mesh according to the Ramirez technique [6]. Follow-up was continued with periodical imaging and standard blood work.

Almost six years after the initial cholecystectomy, the patient presented with an occasionally painful mass in the left axilla. CA19.9 level was 15U/ml, within normal range. An ultrasound showed that the axillary lymph nodes were pathologically enlarged, and a large-needle biopsy was performed. Immunohistochemical staining was positive for cytokeratin (CK) 7, CK19, mucin (Muc) 5ac, and to varying degrees positive for Muc1 (epithelial membrane antigen; EMA). Staining was negative for CDX2, CK20, thyroid transcription factor (TTF)-1, and prostate specific antigen (PSA).

This immunohistochemical profile showed the presence of an adenocarcinoma, most likely arising from the prior gallbladder carcinoma.

**Fig. 1:**
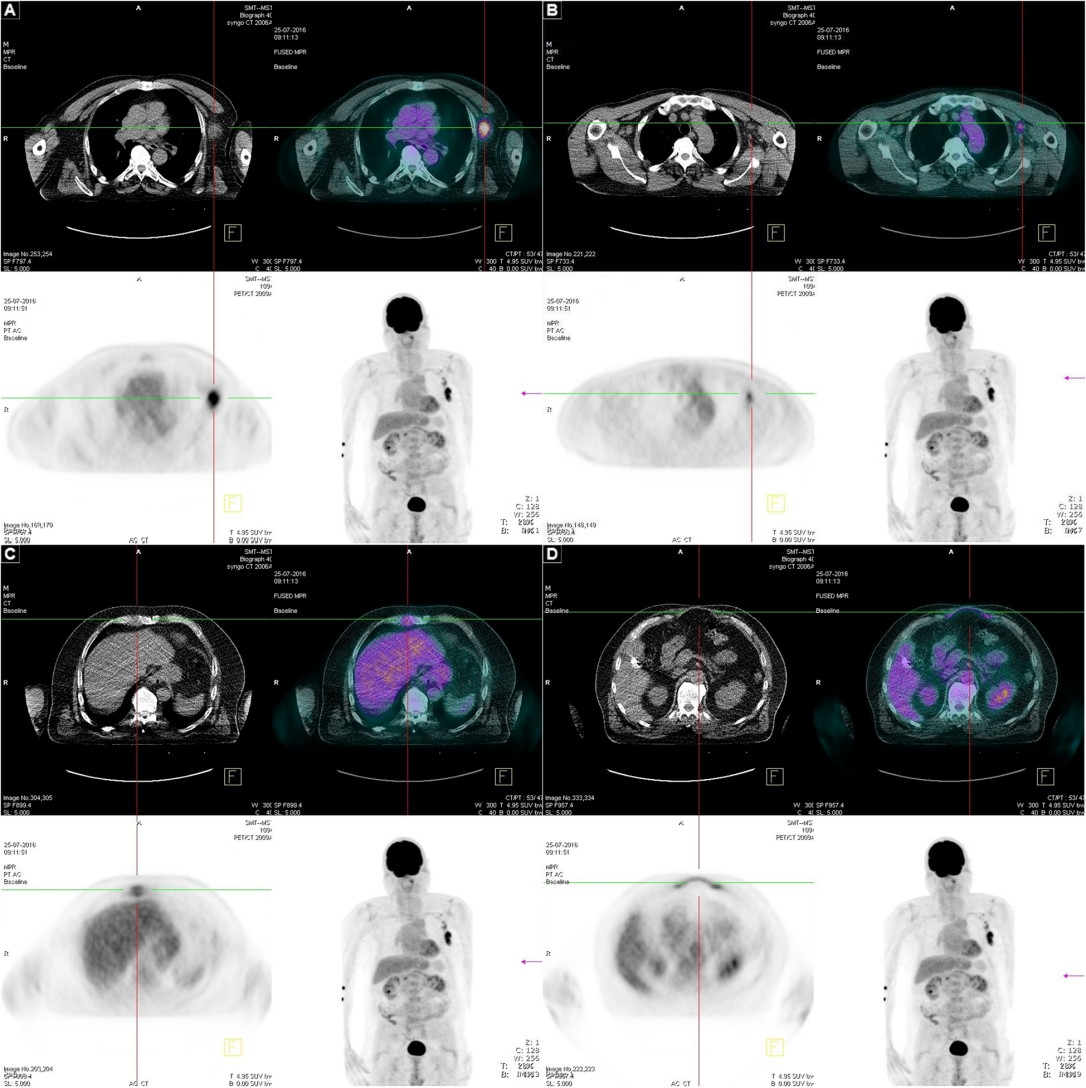
Positron emission tomography–computed tomography of the metastases that occurred almost 6 years after initial cholecystectomy. PET-CT (from the bottom right, clockwise) of the upper body, the axial PET, low-dose CT, and fused PET-CT. **a** axillary metastasis, **b** subpectoral uptake, **c** right parasternal mass, and **d** epigastric port site scar

Subsequently, a positron emission tomography–computed tomography (PET-CT) scan was performed and showed the metastasis in the left axilla (Fig. 1a), and also revealed an elevated subpectoral uptake on the left side (Fig. 1b), with two additional suspicious parasternal masses on the left and right (Fig. 1c), and raised activity in the epigastric port site scar (Fig. 1d). There were no signs of intra-abdominal tumor activity.

Given the extensive dissemination and limited symptoms in the axillary node, we decided against an axillary lymph node dissection and palliative chemotherapy, instead of adopting a wait-and-see policy. Three months later, a new PET-CT scan showed growth of one of the parasternal masses. The patient experienced no progression of symptoms. After multidisciplinary discussion, palliative radiotherapy (5 times 4 gray (Gy)) was performed on the parasternal mass. One month after completion, the patient showed growth of the axillary mass on physical examination. A new CT-scan confirmed growth of the axillary lymph nodes. Subsequently, a new series of radiation was performed (10 times 3 Gy) on the left axilla. Another six months later, palliative chemotherapy was initiated because of disease progression.

The patient died of metastatic disease at the age of 68, approximately 6.5 years after the initial cholecystectomy.

## Discussion and conclusions

Gallbladder carcinomas are relatively rare; their incidence in the Netherlands was 154/100,000 people in 2018, and was most common in patients above the age of 65 [7, 8].

The incidence of port site metastases of gallbladder carcinomas following a laparoscopic cholecystectomy is 8–30%, after a mean period of 4–10 months [1, 4, 9].

The etiology of port site metastases is not entirely clear, but appears to be multifactorial; in addition to the direct contamination of the port site by the instruments or during the removal of the tumor, various other factors have been described (e.g., aerosolization, pneumoperitoneum, immune responses, carbon dioxide, and surgical technique) [9–11].

Multiple perioperative measures during the laparoscopic procedure have been investigated to prevent tumor cell seeding. Among them, port site irrigation using a cytotoxic solution and the closure of the peritoneum at the port sites have been reported to have preventative value, along with careful specimen handling and the use of retractor bags to prevent the mechanical spreading of tumor cells [12].

The curative treatment of gallbladder carcinomas necessitates the radical resection of the malignancy, which seems to be possible in about 27–41% of all cases [13–15]. An additional resection combined with regional lymph node dissection is advised for gallbladder carcinomas invading the subserosal layer or beyond (stage T1b or higher) [14, 16]. However, the excision of port sites to prevent recurrences is not mandatory [17]. Adjuvant therapy is not standard treatment [1].

Although recent studies have shown similar survival rates when comparing laparoscopic surgery with an open approach, the recommended procedure for patients with a suspected gallbladder carcinoma remains a laparotomy, taking into consideration the risk of developing port site recurrences or peritoneal dissemination [1, 7, 18].

Gallbladder carcinomas typically extend locally, invading surrounding structures and lymph nodes in the upper abdomen. Solitary axillary lymph node metastases are extremely rare [19, 20].

It is theorized that axillary lymph node metastases can arise directly from port site recurrences in the abdominal wall in a similar manner to malignancies of the trunk, such as melanoma and breast cancer, which also typically spread to the axillary lymph nodes [20]. The case study presented here, in which subpectoral and parasternal metastases were detected along with the absence of intra-abdominal disease activity, supports this theory.

The life expectancy of patients with truncal lymph node metastases after port site recurrences is short [20].

In the case presented here, we decided against surgical intervention because we suspected the disease had progressed further than the axillary lymph node metastases. It was considered unwise to perform a surgical resection of only part of the disease. Ultimately, there was a short interval between the manifestation of the axillary lymph node metastases and the death of the patient.

Surgical intervention can be considered as a palliative option in exceptional cases; for example, if the metastases are limited to the axillary lymph nodes. If the disease is more progressed, systemic (locoregional) therapy is preferred.

We recommend an aggressive, symptom-based treatment, in which surgery should be limited if other modalities fail.

In conclusion, we presented a patient with incidental gallbladder carcinoma in which axillary lymph node metastases developed 3 years after resection of an epigastric port site recurrence.

Port site recurrences of (incidental) gallbladder carcinoma occur after laparoscopic cholecystectomy, despite preventive perioperative measures. Axillary lymph node metastases can develop from these recurrences. Surgical interventions can be considered in exceptional cases.

## Data Availability

Not applicable.

## Abbreviations

CA 19.9: Carbohydrate antigen 19.9
CK: Cytokeratin
CT: Computed tomography
EMA: Epithelial membrane antigen
Gy: Gray
Muc: Mucin
PET-CT: Positron emission tomography–computed tomography
PSA: Prostate specific antigen
TTF: Thyroid transcription factor
